# Current status and strategies of long noncoding RNA research for diabetic cardiomyopathy

**DOI:** 10.1186/s12872-018-0939-5

**Published:** 2018-10-20

**Authors:** Tarun Pant, Anuradha Dhanasekaran, Juan Fang, Xiaowen Bai, Zeljko J. Bosnjak, Mingyu Liang, Zhi-Dong Ge

**Affiliations:** 1 Department of Medicine, Medical College of Wisconsin, 8701 Watertown Plank Road, Milwaukee, WI 53226 USA; 2 Department of Ophthalmology, Stanford School of Medicine, 1651 Page Mill Road, Stanford, CA 94304 USA; 3 Department of Pediatrics, Medical College of Wisconsin, 8701 Watertown Plank Road, Milwaukee, WI 53226 USA; 4 Department of Cell Biology, Neurology & Anatomy, Medical College of Wisconsin, 8701 Watertown Plank Road, Milwaukee, WI 53226 USA; 5 Department of Physiology, Medical College of Wisconsin, 8701 Watertown Plank Road, Milwaukee, WI 53226 USA; 6 Centre for Biotechnology, Anna University, Chennai, Tamil Nadu India

**Keywords:** Long noncoding RNAs, Diabetic cardiomyopathy, H19, MALAT1, MIAT, SENCR, MT-LIPCAR

## Abstract

Long noncoding RNAs (lncRNAs) are endogenous RNA transcripts longer than 200 nucleotides which regulate epigenetically the expression of genes but do not have protein-coding potential. They are emerging as potential key regulators of diabetes mellitus and a variety of cardiovascular diseases. Diabetic cardiomyopathy (DCM) refers to diabetes mellitus-elicited structural and functional abnormalities of the myocardium, beyond that caused by ischemia or hypertension. The purpose of this review was to summarize current status of lncRNA research for DCM and discuss the challenges and possible strategies of lncRNA research for DCM. A systemic search was performed using PubMed and Google Scholar databases. Major conference proceedings of diabetes mellitus and cardiovascular disease occurring between January, 2014 to August, 2018 were also searched to identify unpublished studies that may be potentially eligible. The pathogenesis of DCM involves elevated oxidative stress, myocardial inflammation, apoptosis, and autophagy due to metabolic disturbances. Thousands of lncRNAs are aberrantly regulated in DCM. Manipulating the expression of specific lncRNAs, such as *H19*, *metastasis-associated lung adenocarcinoma transcript 1,* and *myocardial infarction-associated transcript,* with genetic approaches regulates potently oxidative stress, myocardial inflammation, apoptosis, and autophagy and ameliorates DCM in experimental animals. The detail data regarding the regulation and function of individual lncRNAs in DCM are limited. However, lncRNAs have been considered as potential diagnostic and therapeutic targets for DCM. Overexpression of protective lncRNAs and knockdown of detrimental lncRNAs in the heart are crucial for defining the role and function of lncRNAs of interest in DCM, however, they are technically challenging due to the length, short life, and location of lncRNAs. Gene delivery vectors can provide exogenous sources of cardioprotective lncRNAs to ameliorate DCM, and CRISPR–Cas9 genome editing technology may be used to knockdown specific lncRNAs in DCM. In summary, current data indicate that LncRNAs are a vital regulator of DCM and act as the promising diagnostic and therapeutic targets for DCM.

## Background

Diabetic cardiomyopathy (DCM) refers to diabetes-associated changes in the structure and function of the myocardium that are not directly attributable to other confounding factors such as coronary heart disease or hypertension [1]. It is estimated that DCM occurs in approximately 12% of diabetic patients [2]. DCM is associated with the development of overt heart failure and worse prognosis of diabetic patients [3, 4]. A strategy for prevention and treatment in order to improve the prognosis of DCM has not been established [5–7].

Long noncoding RNAs (lncRNAs) are RNA transcripts longer than 200 nucleotides which, although not having the function of direct coding proteins, can regulate the expression of genes at transcriptional, post-transcriptional, and translational levels [8]. Over the past decade, lncRNAs have received widespread attention as potentially new and crucial players of biological regulation [9, 10]. Their cell-type and tissue-specific expression in health and cardiovascular disease provides the avenue for the diagnosis and treatment of cardiovascular disease [11, 12]. Emerging studies find that lncRNAs are aberrantly regulated in DCM, and impacting the expression of specific lncRNAs is capable of regulating the pathophysiological process of DCM [13–15]. Although the detailed data regarding the role of specific lncRNAs in DCM are limited, they are increasingly identified as a vital regulator of DCM in experimental animals. To get insight into current status of lncRNA research for DCM, we used PubMed and Google Scholar databases to search systemically the published articles that are involved in lncRNAs and DCM. Major conference proceedings of diabetes mellitus and cardiovascular disease occurring between January, 2014 to August, 2018 were also searched to identify unpublished studies that may be potentially eligible. Based on the data obtained from these databases, we present an overview of lncRNA research for DCM. We also discuss the challenges and possible strategies of lncRNAs as diagnostic and therapeutic targets for DCM.

### Diabetes-induced cardiac damage

**Fig. 1:**
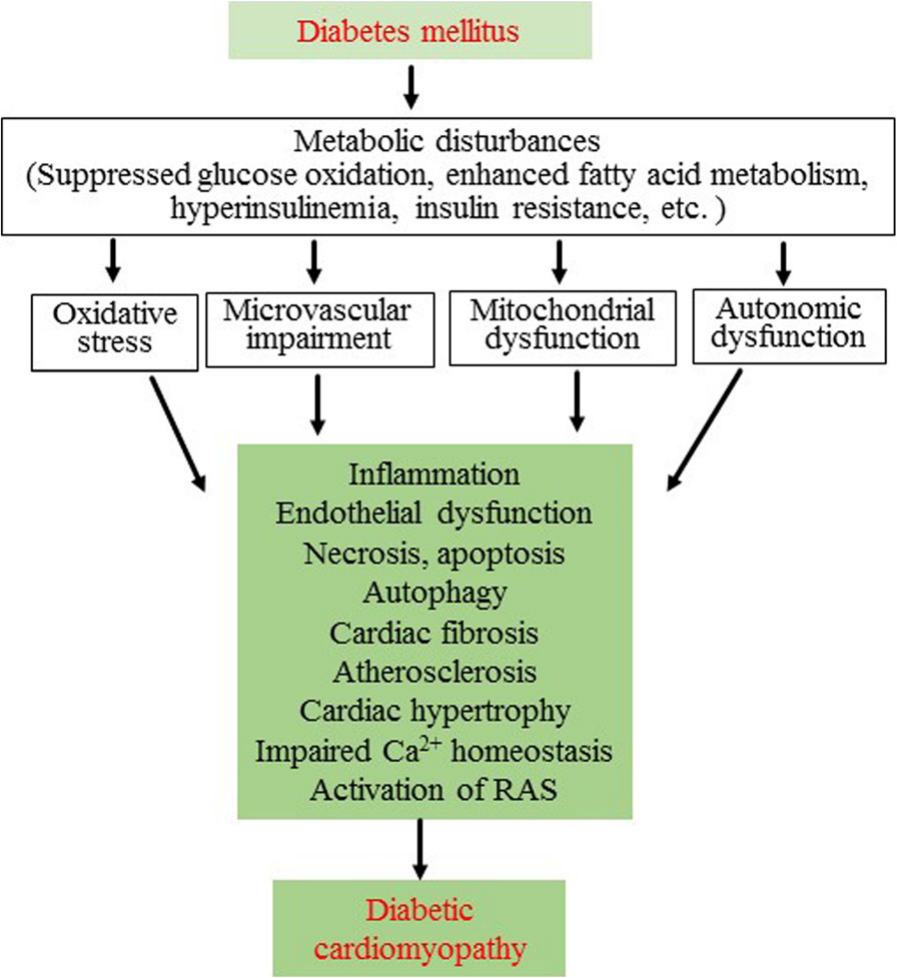
Pathogenesis of diabetic cardiomyopathy. In diabetes mellitus, repressed glucose oxidation, enhanced fatty acid metabolism, hyperinsulinemia, insulin resistance, and accumulation of advanced glycation end-products lead to oxidative stress, microcirculation impairment, mitochondrial dysfunction, and autonomic neuropathy. These pathogenic factors together result in myocardial inflammation, endothelial dysfunction, necrosis, apoptosis, autophagy, fibrosis, athrosclerosis, and cardiac hypertrophy, impair Ca^2+^ homeostasis, and activate the renin-angiotensin system (RAS). Eventually these pathogenic changes in the myocardium impair the diastolic and systolic function of the heart

Diabetes mellitus affects the heart through various mechanisms including metabolic disturbance (suppressed glucose oxidation, enhanced fatty acid metabolism, hyperinsulinemia, insulin resistance, accumulation of advanced glycation end-products, etc.), subcellular component abnormalities, microvascular impairment, and autonomic dysfunction [16, 17]. Eventually myocardium develops local inflammation, coronary arterial endothelial dysfunction, necrosis, apoptosis, autophagy, fibrosis, atherosclerosis, steatosis, and ventricular hypertrophy (Fig. 1) [18, 19]. These pathological changes in the structure, morphology, and function of the heart develop in diabetic patients, especially patients with type 2 diabetes mellitus (T2DM), even without the presence of ischemic heart disease and hypertension, termed diabetic cardiomyopathy (DCM) [1]. It is estimated that DCM occurs in approximately 12% of diabetic patients [2]. Clinical studies indicate that DCM increases the risk of overt heart failure and worsens the prognosis in diabetic patients [3, 4].

Animal models of DCM are critically important for us to advance the understanding of pathogenic mechanisms of DCM and discover new diagnostic and therapeutic targets for DCM. Over the past thirty years, investigators have developed many rodent models of diabetes mellitus and DCM [19, 20]. They are able to provide many advantages in the availability of adequate healthy controls and the absence of confounding factors such as marked differences in age, concomitant pathologies, and pharmacological treatments. Among these models, streptozotocin (STZ)-induced cardiomyopathy of type 1 diabetes mellitus (T1DM) and leptin receptor deficient (db/db)- or leptin deficient (ob/ob)-cardiomyopathy of T2DM are frequently used in the study of lncRNAs [5, 20, 21].

### LncRNAs in the heart

LncRNAs represent one of the most prominent but least understood transcriptome in the heart. According to the NONCODE database (http:www.noncode.org/, version 5), there are 172,216 and 131,697 lncRNA transcripts for humans and mice, respectively. Thousands of lncRNAs have been identified to express abundantly in the myocardial tissues [22–24]. Many of these lncRNAs are dynamically transcribed during the development, differentiation, and maturation of cardiac myocytes [25–27].

LncRNAs have been known to control and regulate the expression of broad ranges of genes in cardiomyocytes [28, 29]. Similar to protein-coding RNAs, individual lncRNAs have specific subcellular distribution that is critical for their functions [30, 31]. Some lncRNAs are enriched in the nucleus and are involved in regulating nuclear processes, such as DNA replication-associated biological processes, mRNA transcription, and RNA processing [23, 32]. In the nucleus, lncRNAs can interact with DNA to form RNA-DNA complexes to reprogram gene expression, act as molecular scaffold, activate or suppress transcription [33, 34]. Other lncRNAs are enriched in the cytoplasm where they can impact protein localization or modulate mRNA stability and translation [35]. LncRNAs can also bind mRNA transcripts to either stabilize or promote translation, cause steric hindrance to block translation (e.g., acting as decoys), regulate RNA splicing and stability, and act as a sponge for microRNAs [36–38]. In the cytoplasm, lncRNAs can interact with proteins to mediate protein trafficking and signaling and impact the function of bound proteins [39].

LncRNA-mediated regulation of gene expression in the heart has been known to involve a variety of mechanisms [40, 41]. Some lncRNAs (for example, cardiac-specific lncRNA *Myheart*) can interact with chromatin remodeling factors to reprogram gene expression [28]. Some lncRNAs (e.g., the lncRNA *Braveheart*) can guide chromatin-modifying complexes to their required genomic destination and serve as docking stations for complex recruitment (acting as scaffolding) [42]. Certain lncRNAs (e.g., the cardiac-enriched lncRNA *Upperhad*) activate transcription of certain genes by guiding transcription factors to their promoters [43, 44]. Particular lncRNAs (e.g., the lncRNA *cardiac autophagy inhibitory factor*) are capable of suppressing transcription by sequestering transcription factors [45]. Some lncRNAs (e.g., the lncRNA *myocardial infarction-associated transcript* [*MIAT*]) can bind to complementary microRNAs (e.g., microRNA-24) via base pairing to sequester them (acting as “microRNA sponges”) [46]. Various lncRNAs (e.g., the lncRNA *metastasis-associated lung adenocarcinoma transcript 1* [*MALAT1*]) can interact with mRNA to regulate their translation and splicing [47, 48]. Other lncRNAs (e.g., *cardiac autophagy inhibitory factor*) can interact with proteins to mediate their trafficking and signaling and regulate the function of bound proteins [45].

LncRNAs play crucial roles in various cardiac diseases [38, 45, 49, 50]. LncRNAs can be targeted to change the physiological function of cardiac myocytes [51, 52]. In cardiac disease, lncRNAs are regulated in a cell type/tissue-specific manner [53, 54]. Manipulating the expression of specific lncRNAs with genetic and pharmacological approaches impacts the severity of myocardial ischemia/reperfusion injury, cardiac hypertrophy, heart failure, and diabetic vascular complications. Thus, certain lncRNAs that are conserved in the heart may have therapeutic potential on various heart diseases [12, 14, 55]. Moreover, some circulating lncRNAs have been proposed to be the biomarker of cardiac disease [56].

### Regulation and function of specific lncRNAs in DCM

**Fig. 2:**
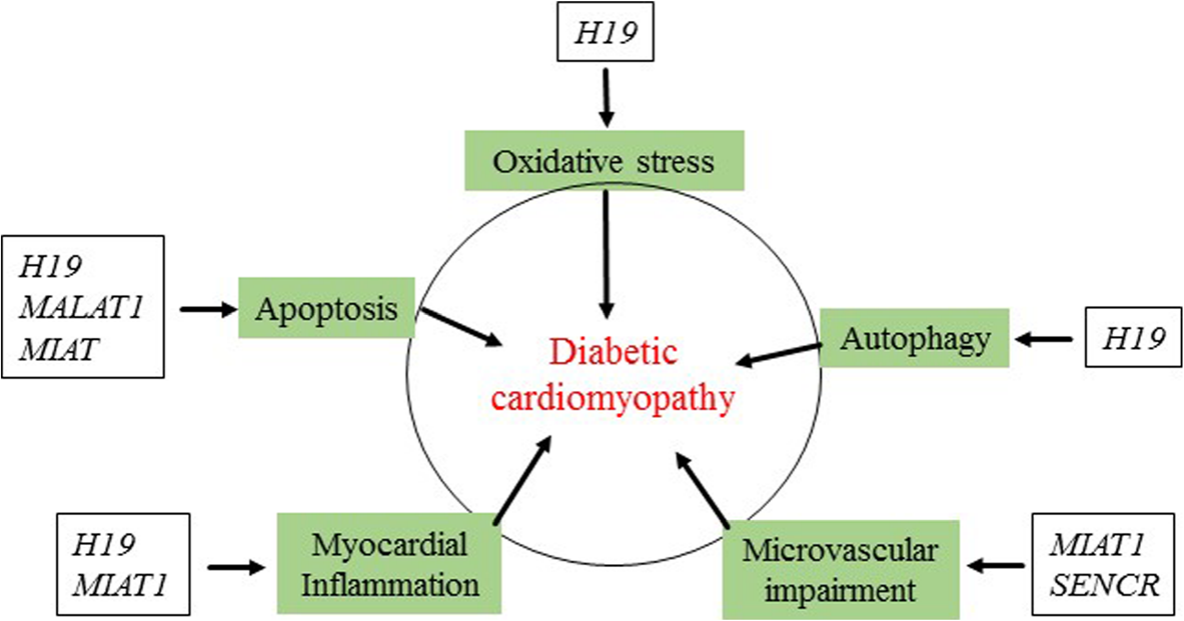
Long noncoding RNAs (lncRNAs) impact the pathophysiological process of diabetic cardiomyopathy. Long noncoding RNAs are regulated in diabetic cardiomyopathy. Changes in the expression of long noncoding RNAs in myocardial tissues influence oxidative stress, myocardial inflammation, cardiomyocyte apoptosis, autophagy, and microvascular impairments. *MALAT1: metastasis-associated lung adenocarcinoma transcript 1; MIAT: myocardial infarction-associated transcript; MT-LIPCAR: the mitochondrially encoded long non-coding cardiac associated RNA; SENCR: smooth muscle and endothelial cell-enriched migration/differentiation-associated long noncoding RNA*

Specific lncRNAs have been identified to express differentially in the heart with DCM [15, 37, 57, 58]. The aberrant expression of specific lncRNAs is associated with the pathophysiological process of DCM, such as oxidative stress, inflammation, apoptosis, myocardial fibrosis, and autophagy (Fig. 2) [15, 37, 57, 58]. Manipulating specific lncRNAs to alter their expression is able to ameliorate DCM [37, 57, 58]. Despite the limited data regarding the regulation and function of specific lncRNAs in DCM, lncRNAs are considered as a promising target/candidate for the treatment and diagnosis of DCM. In this section, we discuss several of the lncRNAs that may have a good potential as a target/candidate for the treatment and diagnosis of DCM (Table 1).

**Table 1:** Regulation and function of specific long noncoding RNA in DCM

LncRNAs	Models	Species	Regulation during DCM	Function in DCM	References
*H19*	STZ-included T1DM	Rats	Down	Suppress oxidative stress, inflammation, apoptosis, and autophagy	[57, 58]
*MALATI*	STZ-included T1DM	Rats	Up	Suppress inflammation and apoptosis	[15, 84]
*MIAT*	STZ-included T1DM	Rats	Up	Decrease apoptosis	[37]
*SENCR*	db/db T2DM	Mice	Down	Promote proliferation and migration of smooth muscle cells	[87]
*MT-LIPCAR*	T2DM	Humans	Down	Not available	[13]

*DCM* diabetic cardiomyopathy, *LncRNAs* long noncoding RNAs, *STZ* streptozocin, *MALATI* metastasis-associated lung adenocarcinoma transcript 1, *MIAT* myocardial infarction-associated transcript, *SENCR* smooth muscle and endothelial cell-enriched migration/defferentiation-associated long noncoding RNA, *MT-LIPCAR* the mitochondrially encoded long non-coding cardiac associated RNA

#### H19

*H19* is a 2.3-kb lncRNA which is transcribed from H19/insulin-like growth factor-II (IGF2) genomic imprinted cluster located on human chromosome 11p15.5 (syntenic to mouse chromosome 7) [59]. *H19* and IGF2 genes are expressed in a monoallelic fashion from the maternal and paternal chromosomes, respectively [60, 61]. *H19* is transcribed by a polymerase II [62]. *H19* transcripts start from the blastocyst stage and reach a high level in the tissues of endodermal, mesodermal, and ectodermal origins [63]. After the birth, *H19* expression will be inhibited in most of mammalian tissues [64]. However, *H19* remains in high accumulation in mature myocardium of both mice and humans possibly due to enhanced RNA stabilization during cardiomyocyte differentiation [65]. Both primary sequence and secondary structures of *H19* show a great extent of conservation among mammals [66].

*H19* has recently been identified as an important regulator of the cardiomyopathy of T1DM in experimental rats [57, 58]. Sprague-Dawley rats injected with STZ developed the cardiomyopathy of T1DM with decreased expression of cardiac *H19* [57, 58]. Overexpression of *H19* in myocardial tissues caused decreases in oxidative stress, inflammation, apoptosis, and autophagy, leading to the amelioration of DCM [57, 58].

*H19* serves as template for microRNA-675 expression from H19 first exon [67, 68]. Since microRNA-675 has multiple targets in diverse signaling pathways, *H19* is able to regulate a number of biological processes via microRNA-675. For example, the *H19*/microRNA-675 reduces high glucose-induced apoptosis by targeting voltage-dependent anion channel 1 which is a critical protein required for the mitochondria-mediated apoptosis [58, 69]. In addition, by down-regulating GTP-binding protein Di-Ras-3, the *H19*/microRNA-675 promotes the phosphorylation of the mechanistic target of rapamycin and inhibits activated autophagy in cardiomyocytes exposed to high glucose [57]. Another pattern of *H19* exerting its function is through interacting with proteins and microRNAs. H19 is capable of being folded into a special secondary structure, which allows it to serve as a platform and collect relative proteins [70]. Multiple proteins have been identified to associate with H19, including the RNA binding proteins, KH-type splicing regulatory proteins, inner membrane protease 1, the Hu family of RNA-binding proteins, heterogeneous nuclear ribonucleoprotein U, polypyrimidine tract-binding protein 1, the DNA/chromatin modification factors, S-adenyl-L-homocysteine hydrolase, polycomb repressive complex 2, p53, and isoleucyl tRNA synthetase of mitochondria [68]. These proteins are actively involved in a wide variety of physiological and pathological processes, such as RNA metabolism, gene transcription, and epigenetic modification [68]. MicroRNAs are another group of partners that are essential for H19 to exert its function. It is evident that *H19* interacts with Let-7, microRNA-138, microRNA-200a, microRNA-106a, and microRNA-141 [68].

IGF2 proteins are an important growth factor during pregnancy, where they promote both fetal and placental growth [71, 72]. However, the overexpression of IGF2 and its receptors in acute hyperglycemia and diabetes is associated with the progression of DCM by triggering cardiac hypertrophy and apoptosis [73]. The effect of *H19* overexpression on the levels of myocardial IGF2 in adults remains unclear. In embryos, the overexpression of *H19* results in a decrease in IGF2 expression due to a *cis* effect of the *H19* locus on the adjacent IGF2 gene [74]. It is reasonably believed that IGF2 levels are decreased too in *H19*-overexpressing animals, and decreased IGF2 contributes to the beneficial effects of *H19* overexpression on DCM.

In summary, cardiac *H19* is downregulated in DCM, and transgenic overexpression of *H19* improves DCM by attenuation of myocardial oxidative stress, inflammation, apoptosis, and autophagy.

#### MALAT1

*MALAT1* is a nuclear transcript localized to the nuclear speckles, a nuclear domain for storage and/or the sites of pre-mRNA splicing [75]. Pre-mRNAs splicing is a pivotal step between transcription and translation of most eukaryotic mRNAs [76]. *MALAT1* interacts with several serine/arginine proteins, such as serine/arginine-rich splicing factors and spliceosomal proteins, to regulate pre-mRNA splicing [77–79]. In addition, *MALAT1* is involved in nuclear organization and epigenetic modulation of gene expression [80, 81]. *MALAT1* was abundantly expressed in cardiac myocytes and highly conserved across mammalian species [82, 83]. In the rat cardiomyopathy of T1DM induced by streptozotocin, MALAT1 in myocardial tissues was up-regulated [15, 84]. The knockdown of *MALAT1* with the small interfering RNA to attenuate the expression of *MALAT1* in diabetic hearts significantly attenuated inflammation and apoptosis and improved DCM [15, 84]. Thus, the upregulation of *MALAT1* represents a critical pathogenic mechanism for DCM.

In short, cardiac *MALAT1* is upregulated in DCM, and the knockdown of *MALAT1* improves DCM by attenuation of myocardial inflammation and apoptosis.

#### MIAT

*MIAT* is first identified to be associated with myocardial infarction in a genome-wide association study in 2006 [85]. Before that, *MIAT* was also known as *RNCR2*, 2 *AK02836* or *GOMAFU*. *MIAT* may function as a competing endogenous RNA to upregulate the expression of death-associated protein kinase-2 by sponging miR-22-3p, which consequently leads to the apoptosis of cardiac myocytes [37]. Like *MALAT1*, the expression of cardiac *MIAT* was significantly upregulated in Sprague-Dawley rats with the cardiomyopathy of T1DM [37]. The knockdown of *MIAT* with *MIAT*-shRNA resulted in improvement of DCM and reduction of apoptosis of cardiac myocytes [37]. The inhibitory effect of *MIAT* knockdown on apoptosis is attributed to a decrease in the expression of death-associated protein kinase-2. Taken together, the upregulation of cardiac *MIAT* contributes to the pathogenesis of DCM.

#### Smooth muscle and endothelial cell-enriched migration/differentiation-associated long noncoding RNA (SENCR)

*SENCR* is a vascular cell-enriched lncRNA [86]. It promotes the proliferation and migration of smooth muscle cells through regulation of forkhead box protein O1 and transient receptor potential cation channel 6. However, *SENCR* was down-regulated in T2DM db/db mice and in vascular smooth muscle cells exposed to high glucose [87]. The overexpression of *SENCR* reversed the inhibitory effect of high glucose on the proliferation and migration of mouse vascular smooth muscle cells. Both clinical and experimental studies indicate that impaired vascular smooth muscle cells by diabetes and high glucose contribute to the increased incidence of DCM [88]. Although there are no reports about the direct impacts of *SENCR* on DCM, the downregulation of cardiac *SENCR* may contribute to the pathogenesis of DCM.

#### The mitochondrially encoded long non-coding cardiac associated RNA (MT-LIPCAR)

*MT-LIPCAR* (*uc022bqs.1*, Gene ID: 103504742*)* is a 781-nucleotide lncRNA which is possibly transcribed from mitochondrial DNA [89]. It can cross the membrane barrier and is released into the circulation. Although there are a large number of RNase in plasma [90], *MT-LIPCAR* is stable in blood serum/plasma [13, 49, 91]. Recently, de Gonzalo-Calvo et al. analyzed lncRNAs derived from the serum of 48 patients with cardiomyopathy of T2DM and 12 healthy volunteers [13]. *MT-LIPCAR* levels in plasma were positively associated with left ventricular diastolic dysfunction. Moreover, *MT-LIPCAR* was strongly correlated with waist circumference, plasma fasting insulin, subcutaneous fat volume, and high-density lipoproteins-C. Collectively, *MT-LIPCAR* may be an independent predictor of diastolic dysfunction in T2DM patients with DCM [13].

In the clinic, the specific diagnosis of DCM is difficult, since the patients are asymptomatic in the early and middle stages and may concomitantly suffer from ischemic heart disease or hypertension during the late stage [7, 92, 93]. The significant increase in the levels of specific lncRNAs in serum/plasma of patients with DCM, such as *MT-LIPCAR*, could make lncRNAs specific biomarkers for the diagnosis and prognosis of DCM. A clinical trial recently suggests that *MT-LIPCAR* in plasma may serve as a promising biomarker of DCM [13]. The value of *MT-LIPCAR* and other circulating lncRNAs as diagnostic and prognostic markers in DCM needs to be validated. Large multicenter randomized, controlled trials with *MT-LIPCAR* need to be conducted in patients with DCM.

#### Antisense non-coding RNA in the INK4 locus (ANRIL)

*ANRIL* [alias *cyclin dependent kinase inhibitor 2B antisense RNA 1 (CDKN2B-AS1*) and *P15 antisense RNA* (*P15AS*)] is a 3.8 kb lncRNA transcribed from the short arm of human chromosome 9 on p21.3 [94]. *ANRIL* and the adjacent protein coding genes, *cyclin dependent kinase inhibitor 2A (CDKN2A)* and *cyclin dependent kinase inhibitor 2B (CDKN2B), locate on* chromosome 9p21 [95]. The *CDKN2A* gene encodes several transcripts/proteins, the p16 protein of which functions as inhibitors of cyclin-dependent kinase 4 [96, 97]. The *CDKN2B* gene encodes cyclin-dependent kinase 4 inhibitor B that functions as a cell growth regulator that control cell cycle G1 progression [98]. *ANRIL is an antisense of the CDKN2B gene and* is transcribed by RNA polymerase II and spliced into multiple linear and circular isoforms in a tissue-specific manner [99]. *ANRIL* is capable of recruiting polycomb group proteins to modify the epigenetic chromatin state and binding to a site or sequence to regulate gene expression [100]. It is well known to know that single nucleotide polymorphisms in the human chromosome 9p21 locus are associated with diabetes, cardiovascular disease, and multiple cancers [101–106]. Recent studies have identified *ANRIL* as a highly susceptible region for T2DM, coronary artery disease, and hypertension [107]. Although there is no report regarding the role of *ANRIL* in DCM, it is reasonably believed that *ANRIL* might be involved in the pathogenesis of DCM.

In summary, ANRIL is a potential candidate that is associated with the pathogenesis of DCM.

### Challenges and potential strategies of lncRNA research for DCM

LncRNAs may be a promising target and/or candidate as biomarkers of DCM diagnosis and for the treatment of DCM. However, at present the function and regulation of thousands of lncRNAs in DCM are still ambiguous. Recently, we performed a systemic microarray-based analysis of the cardiac expression profiles of lncRNAs in T2DM db/db mice on a genetic background of C57BL/6 mice with and without DCM. Among the 23,578 lncRNAs identified, 1479 were differentially expressed in the myocardium of db/db mice between with DCM and without DCM [108]. These results suggest that at least 1479 lncRNAs might be involved in DCM in obese type 2 db/db mice. Determining the individual functionality of these lncRNAs is important for good understanding of cardiac developmental biology and DCM. For the study of individual lncRNAs in DCM, the following questions should be considered: Do lncRNAs contribute to the pathogenesis of DCM? How stable are the lncRNAs in circulation? Is their stability altered in diabetes mellitus and cardiac dysfunction? Are lncRNAs toxic? What are the pharmacokinetics of the lncRNAs? Answering these questions will be important as we study the individual lncRNAs and their role in diagnosis and treatment of DCM.

Some lncRNAs are protective to DCM, such as *H19*. These lncRNAs are down-regulated in DCM [57], and their overexpression in the heart is considered as a therapeutic strategy for DCM [58]. Owing to the length of lncRNA molecules their overexpression in cardiomyocytes is a complicated matter. Moreover, the long modified transcript is difficult to cross the membrane barrier. Thus, its efficient in vivo delivery would be difficult. Recent studies have reported that gene delivery vectors are capable of provide exogenous expression of the desired lncRNAs [38]. Utilization of gene delivery vectors, like engineered adeno-associated virus, is an alternative approaches to increase the expression of protective lncRNAs in the heart to ameliorate DCM.

Up-regulation of detrimental lncRNAs in DCM, such as *MALAT1* and *MIAT*, could make them promising therapeutics targets for DCM [109]. However, in vivo inhibition of detrimental lncRNAs is a challenge mainly due to their short half live as they are easily degraded by nucleases in bio fluids and the length of lncRNA transcripts. At present, the approaches which are used to manipulate lncRNAs in vivo include mainly the use of small interfering RNAs, antisense oligonucleotides, and the 5′ and 3′ end-modified antisense oligonucleotides, GapmeRs [53, 110]. Each of these approaches have their own advantages and disadvantages. Small interfering RNAs specifically bind to complementary sequences and inhibit the expression of lncRNA targets [111, 112]. Antisense oligonucleotides are capable of targeting specific genes or transcripts directly through Watson-Crick base pairing, and they thus can reduce the levels of lncRNAs of interest [113]. Locked nucleic acid GapmeRs can modulate target lncRNA expression, block lncRNA activity, or induce enzyme-mediated degradation [53, 114]. Despite the potential therapeutic value of small interfering RNAs, antisense oligonucleotides, and GapmeRs in treating human disease, the effects of these approaches may have varied efficacy within the cell due to poor accessibility. Many studies have made use of antisense oligonucleotides to knockdown lncRNAs successfully for functional studies in mice or rats [115–117]. Compared with small interfering RNAs, antisense oligonucleotides are able be a better approach since cytoplasmic lncRNAs are efficiently ablated using small interfering RNA. To inhibit upregulated lncRNAs that show co-localization, the hybrid approach works the best [111].

Some lncRNAs are refractory to inhibition by either antisense oligonucleotides or small interfering RNAs. This may be related to the subcellular localization of the lncRNAs, which is not accessible to either RNase H or the interfering RNA machinery [111]. Another cause may be that the lncRNAs are highly structured or blocked due to excessive protein binding or hybridizing to other cellular nucleic acids. To overcome these hurdles, it is necessary to produce a high-throughput method to delete lncRNAs. Emerging studies suggest that CRISPR–Cas9 genome editing technology is able to quickly and effectively delete lncRNAs [118, 119]. Despite no reports about the utilization of CRISPR–Cas9 genome editing technology in DCM, this technology is a potential tool to delete the lncRNAs of interest and modulate the expression of lncRNAs in DCM.

In short, both overexpression of protective lncRNAs and knockdown of detrimental lncRNAs in the heart are crucial for defining the role and function of the lncRNAs of interest in DCM. Either approach is technically challenging due to the length, short life, and location of the lncRNAs of interest. In addition to traditional utilization of small interfering RNAs, antisense oligonucleotides, and GapmeRs to inhibit the lncRNAs of interest, CRISPR–Cas9 genome editing technology is a potential tool to knockdown specific lncRNAs.

## Conclusions

LncRNAs play vital roles in the pathogenesis of DCM. Manipulating specific lncRNAs with pharmacological and genetic approaches to alter their expression impacts the development of DCM. In spite of limited data of specific lncRNAs in DCM, they are the potential targets/candidates for DCM. The future research needs to elucidate the regulation, function, and action mechanisms of more lncRNAs in the pathogenesis of DCM to search potential targets/candidates as diagnostic biomarkers of DCM and potential treatment of DCM.

## Abbreviations

ANRIL: Antisense non-coding RNA in the INK4 locus
DCM: Diabetic cardiomyopathy
IGF2: Insulin-like growth factor-II
lncRNAs: Long noncoding RNAs
MALAT1: Metastasis-associated lung adenocarcinoma transcript
MIAT: Myocardial infarction-associated transcript
MT-LIPCAR: The mitochondrially encoded long non-coding cardiac associated RNA
SENCR: Smooth muscle and endothelial cell-enriched migration/differentiation-associated long noncoding RNA
STZ: Streptozotocin
T1DM: Type 1 diabetes mellitus
T2DM: Type 2 diabetes mellitus
